# Seeing hormones in action: High-resolution gibberellin dynamics in nodules

**DOI:** 10.1093/plcell/koae216

**Published:** 2024-07-23

**Authors:** Min-Yao Jhu, Thomas B Irving

**Affiliations:** Assistant Features Editor, The Plant Cell, American Society of Plant Biologists; Crop Science Centre, Department of Plant Sciences, University of Cambridge, Cambridge, UK; Crop Science Centre, Department of Plant Sciences, University of Cambridge, Cambridge, UK

Imagine watching plant hormones like gibberellin (GA) orchestrate developmental processes from seed germination to stem elongation at the cellular level in real time. Using Förster Resonance Energy Transfer (FRET) biosensors, which permit single-cell GA measurements in vivo, researchers have unveiled intricate dynamics of GA in the hypocotyls of Arabidopsis (*Arabidopsis thaliana*) (Griffiths et al. 2024) and the root nodules of *Medicago truncatula* (Drapek et al. 2024). This visualization illustrates how GA accumulates and regulates cell division and elongation in response to environmental cues across different tissues.

FRET biosensors measure molecular interactions by alterations in the energy transfer between fluorescent proteins due to changes in protein shape during ligand interaction. The first FRET biosensor for GA, nuclear-localized Gibberellin Perception Sensor 1 (nlsGPS1), was developed in 2017 and revealed cellular GA dynamics during seedling growth induced by light and darkness (photomorphogenesis and skotomorphogenesis) (Rizza et al. 2017, 2021). Although GPS1 has a high affinity to bioactive GA, slow reversibility limited its use in studying rapid GA depletion dynamics. Moreover, plants expressing nlsGPS1 showed hyposensitivity to the GA biosynthesis inhibitor paclobutrazol, indicating nlsGPS1 interfered with endogenous GA signalling (Rizza et al. 2017; Griffiths et al. 2024). Griffiths et al. therefore developed the second-generation GA biosensor, GPS2, via charge exchange between the binding domains, which reduced interaction with endogenous signaling and enhanced reversibility (Fig. 1A). GPS2 enabled the discovery of a rapid GA depletion during the switch to photomorphogenesis, which is critical for limiting further cell elongation (Griffiths et al. 2024).

**Figure 1.**
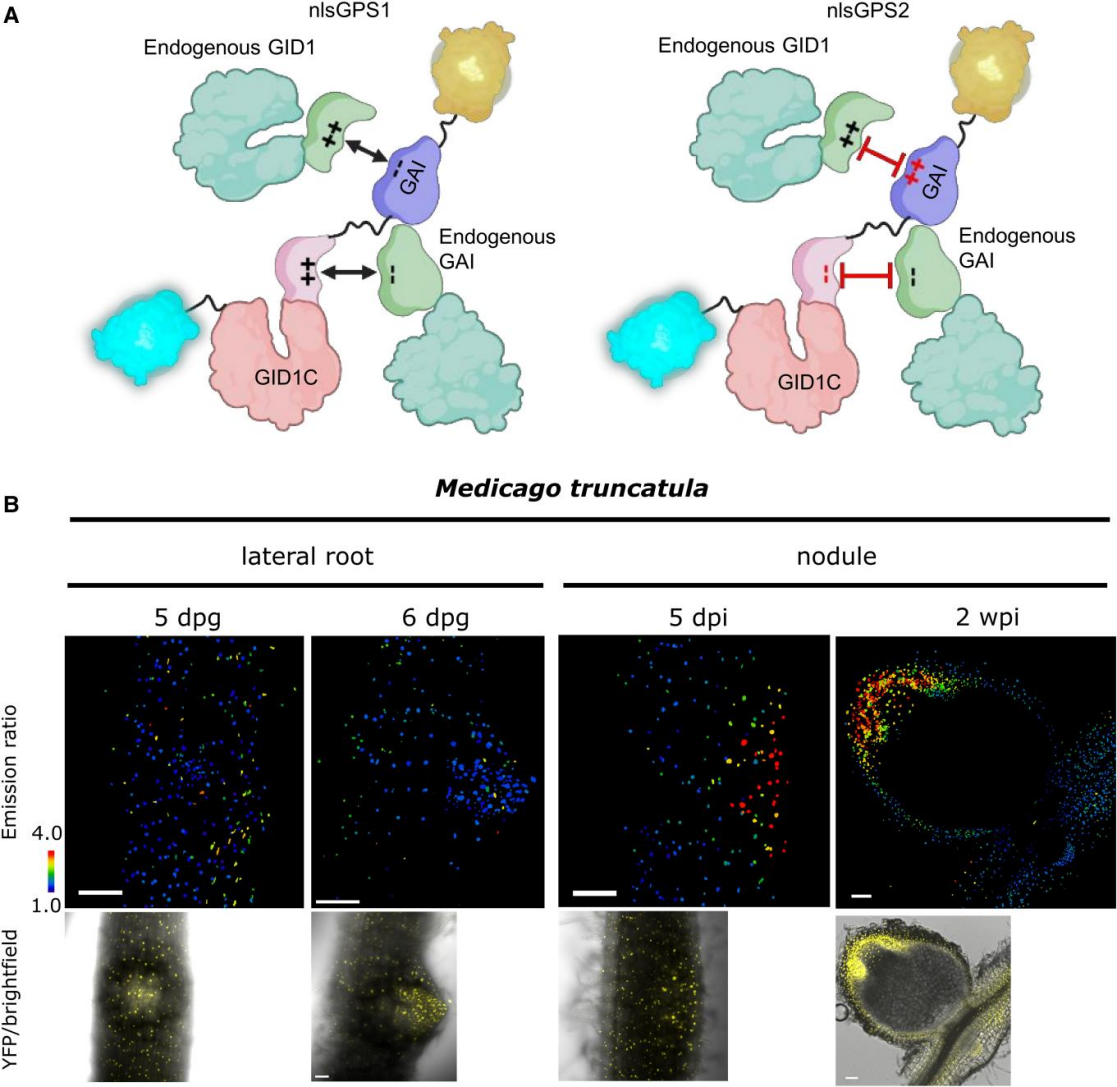
nlsGPS2, a second-generation FRET biosensor for GA. **A)** Diagram illustrating the engineering of nlsGPS2. Key modifications include the exchange of 2 pairs of charged residues between the GID1C and DELLA interacting interfaces in the sensor to reduce its ability to dimerize with endogenous GID1 and DELLA proteins. **B)** Spatial distribution of GA as visualized by nlsGPS2 as a FRET ratio (1/blue = low GA, 4/red = high GA) and control fluorescence shown in yellow/brightfield. Bar = 100 *µ*m; dpg = days post germination; dpi = days post infection. Adapted from Griffiths et al. (2024), Figure 5, and Drapek et al. (2024), Figure 5.

Using GPS2, Drapek et al. visualized the dynamic spatial distribution of GA during nodule formation in *M. truncatula*. During early nodule development, GA levels peak in the cortical cells (Fig. 1B), facilitating cell division to build the new organ while remaining low in the outer cortex and epidermis (Drapek et al. 2024). This targeted GA localization in only the inner cortex supports the accumulation of DELLA proteins in the outer cell layers, which are positive regulators of rhizobia infection and are known to be degraded by GA (Fonouni-Farde et al. 2016).

On the other hand, in the mature nodule, GA accumulated in the nodule meristem (Fig. 1B), correlated with dividing cells (Drapek et al. 2024). Manipulating GA levels influenced nodule size, with increased GA levels promoting larger nodules and reduced GA leading to smaller nodules, confirming this local GA accumulation determines the symbiotic organ size (Drapek et al. 2024). These findings indicate that GA's influence extends beyond regulating rhizobial infection via DELLA degradation. The sensor was not observed in the center of the mature nodule, likely because the oxygen-sensitive nitrogenase requires tightly regulated oxygen levels (Rutten and Poole 2019), and the fluorophores in nlsGPS2 need oxygen to mature. Consequently, the authors cannot conclude GA's role in the fixation zone, a limitation that may extend to the use of nlsGPS2 in studying other anoxic conditions like flooding.

Although lateral roots and nodules shared overlapping developmental programs, lateral roots exhibit minimal GA accumulation (Fig. 1), highlighting its specific role in nodulation rather than general organ development (Drapek et al. 2024). GA accumulation also required several key transcriptional regulators involved in nodule development and identity, such as the transcription factor NODULE INCEPTION (NIN) and its downstream elements LIGHT-SENSITIVE SHORT HYPOCOTYL proteins 1 and 2 (LSH1/2), and NODULE ROOT 1 and 2 (NOOT1/NOOT2). In *noot* and *lsh* mutants, the expected GA patterning is lost during nodule maturation, corresponding with nodules reversion to lateral roots. These findings confirm GA as a positive regulator of cortical cell division needed for nodule initiation and growth, and indicate a possible role in the maintenance of organ identity (Drapek et al. 2024).

FRET biosensors provide a real-time, cell-specific view of GA dynamics across various species and tissues. Studies in hypocotyls and nodules used nlsGPS2 to resolve GA dynamics at the cellular level, offering insights into GA's regulation during developmental processes (Griffiths et al. 2024; Drapek et al. 2024). These findings highlight GA's role in determining cell size, cell identity, and organ size and demonstrate how anabolism and catabolism alter hormone concentration in response to environmental cues, like light and symbiont, within hours (Griffiths et al. 2024; Drapek et al. 2024). These results exemplify how cutting-edge tools can offer new insights into hormonal regulation, opening novel avenues for enhancing plant growth and productivity. However, it is important to note that hormone concentration may not always directly translate into signaling activity, since cells can modulate responses by varying the expression of signaling proteins. As a suggestion for future work, combining nlsGPS2 with another sensor, such as a promoter: reporter or DELLA degron sensor (ratiometric fluorescent reporters which monitor protein degradation rate), could help ensure that measured hormone concentrations correspond to cellular responses.

## References

[koae216-B1] Drapek C , Rizza A, Mohd-Radzman NA, Schiessl K, Dos Santos Barbosa F, Wen J, Oldroyd GED, Jones AM. Root nodulation cellular gibberellin dynamics are distinct from lateral roots and governs nodule development, morphology, and maturation. Plant Cell. 2024:36(10):4442–4456. 10.1093/plcell/koae201 PMC11449112 39012965

[koae216-B2] Fonouni-Farde C , Tan S, Baudin M, Brault M, Wen J, Mysore KS, Niebel A, Frugier F, Diet A. DELLA-mediated gibberellin signalling regulates Nod factor signalling and rhizobial infection. Nat Commun. 2016:7(1):12636. 10.1038/ncomms12636 27586842 PMC5025792

[koae216-B3] Griffiths J , Rizza A, Tang B, Frommer WB, Jones AM. Gibberellin perception sensors 1 and 2 reveal cellular GA dynamics articulated by COP1 and GA20ox1 that are necessary but not sufficient to pattern hypocotyl cell elongation. Plant Cell. 2024:36(10):4426–4441. 10.1093/plcell/koae198 PMC11449061 39039020

[koae216-B4] Rizza A , Walia A, Lanquar V, Frommer WB, Jones AM. In vivo gibberellin gradients visualized in rapidly elongating tissues. Nat Plants. 2017:3(10):803–813. 10.1038/s41477-017-0021-9 28970478

[koae216-B5] Rizza A , Tang B, Stanley CE, Grossmann G, Owen MR, Band LR, Jones AM. Differential biosynthesis and cellular permeability explain longitudinal gibberellin gradients in growing roots. Proc Natl Acad Sci. 2021:118(8):e1921960118. 10.1073/pnas.1921960118 33602804 PMC7923382

[koae216-B6] Rutten PJ , Poole PS. Oxygen regulatory mechanisms of nitrogen fixation in rhizobia. Adv Microb Physiol. 2019:75:325–389. 10.1016/bs.ampbs.2019.08.001 31655741

